# Climate Change, Conflict, and Resource Extraction: Analyses of Nigerian Artisanal Mining Communities and Ominous Global Trends

**DOI:** 10.5334/aogh.3547

**Published:** 2022-03-08

**Authors:** Casey Bartrem, Ian von Lindern, Margrit von Braun, Simba Tirima

**Affiliations:** 1 TerraGraphics International Foundation, Moscow Idaho US; 2 University of Idaho, Moscow Idaho US; 3 Médecins Sans Frontières, Abuja, NG

## Abstract

**Background:**

The 2010 lead poisoning outbreak that claimed the lives of more than 400 children in artisanal gold mining villages in Zamfara, Nigeria is the tragic result of high gold prices, a geologic anomaly, and processing of ores in residential areas. Today, these villages face a growing crisis related to conflict and climate change. While the situation in Zamfara is unparalleled in many ways, the interactions between climate change, conflict, and mining consistently overlap a global scale. The scope of this analysis extends beyond the Nigerian crisis.

**Objectives:**

Understanding the complexities of challenges faced in Zamfara provides insight into how these issues impact vulnerable communities globally, and which strategies should be considered to solve this wicked problem.

**Methods:**

Analysis of the relationships between climate change, conflict, and mining in Zamfara and globally via literature review and examination of current events in the Sahel region.

**Findings:**

Supporting healthy artisanal mining communities, as was prioritized in Zamfara, must be a focus of environmental, health, and mineral management policies. This includes the consideration of multiple environmental health challenges, the protection of vulnerable groups, government-supported formalization programs, and meaningful involvement of local leadership in developing, implementing, and sustaining intervention strategies to enshrine ASM as a poverty reduction, climate change adaptation strategy.

**Conclusions:**

Rapidly rising metal prices and demand will continue to fuel environmental health crises associated with mining. Given Africa’s growing role in the global mineral economy and the massive number of subsistence communities who will continue to be impacted by climate change, strategies that support responsible artisanal mining are both a necessity for preventing future health crises and an opportunity for promoting regional stability and peace.

## Introduction

A tragic lead poisoning outbreak claimed the lives of 400 children and severely poisoned 30,000 more people in isolated artisanal gold mining villages in Zamfara State, Nigeria in 2010 [1,2,3]. In the 11 years since the outbreak was discovered, the Nigerian government and domestic and international organizations have collaborated on an environmental health response that includes medical treatment, environmental remediation, monitoring, institutional controls, and safer mining practices. Despite the severity and unprecedented nature of the crisis in Nigeria, project partners have made remarkable progress in reducing lead-related morbidity and mortality rates among children [1,4]. Yet along with successes, both the communities and the project face increasing challenges from climate-related conflicts in the region [5].

**Figure 1:**
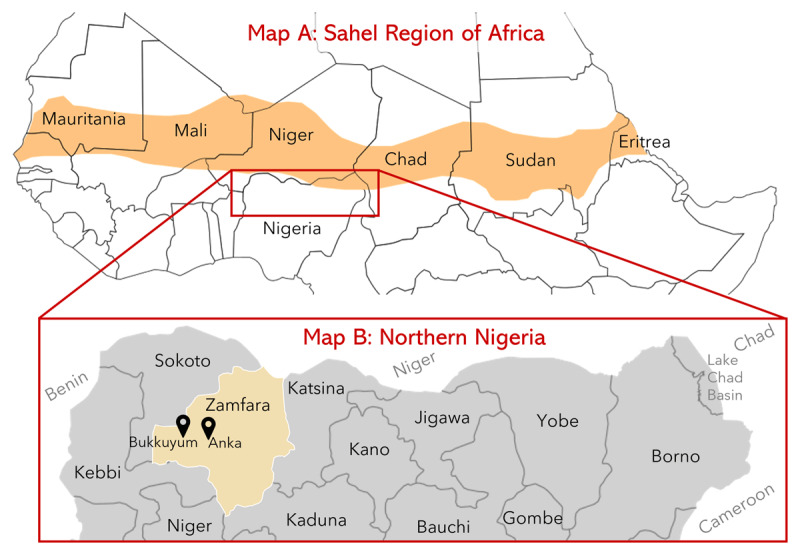
Map A shows the northern Africa region, with the Sahel in orange. Map B highlights Zamfara State and the two Local Government Areas (Anka and Bukkuyum) where artisanal mining communities have been impacted by lead poisoning.

**Figure 2:**
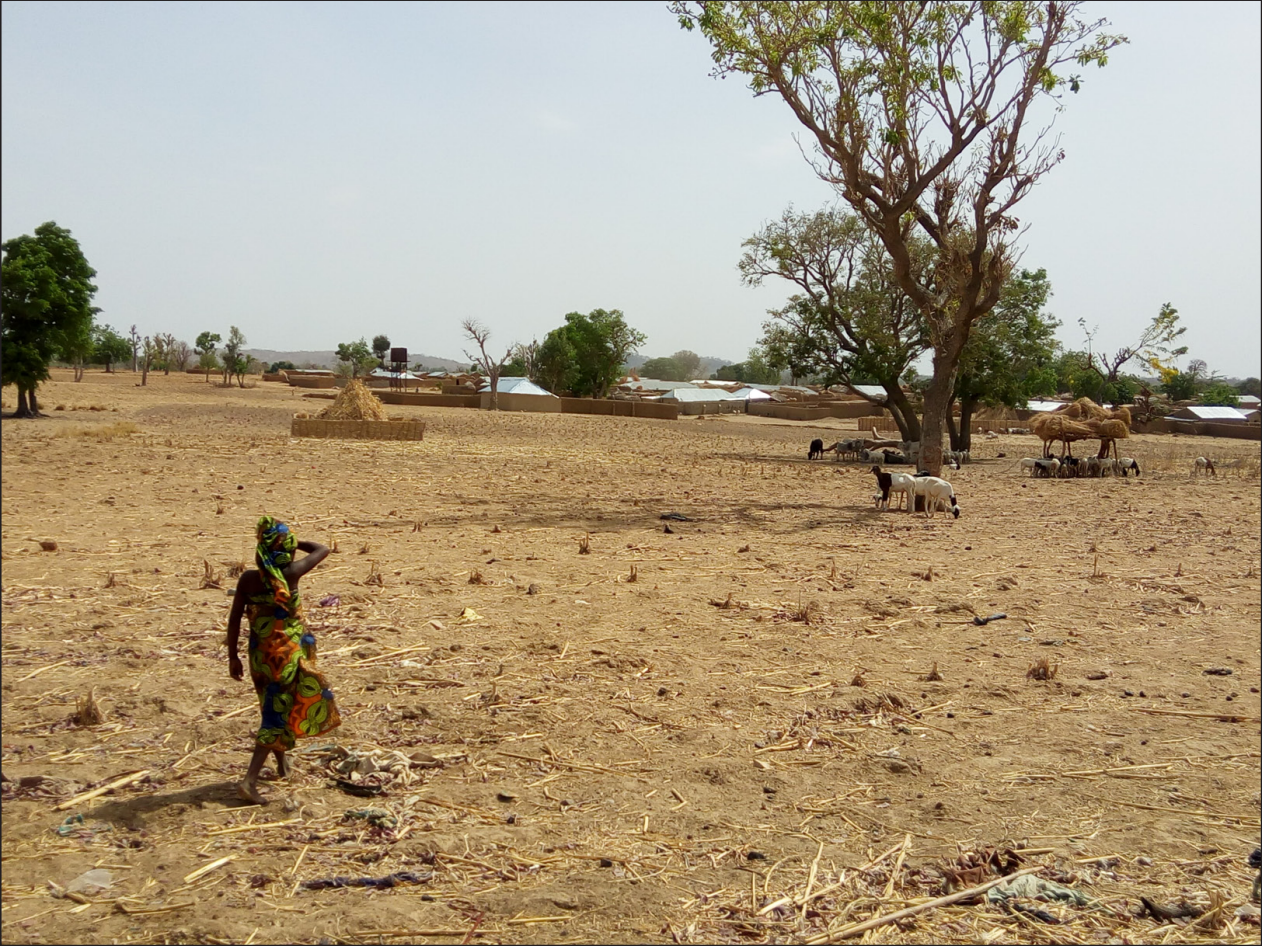
A child walks home on the outskirts of Abare Village, Zamfara State in 2011 (photo credit: Casey Bartrem Casey Bartrem/TIFO).

Zamfara is located on the southern border of the Sahel, a semi-arid biogeographic zone that separates the Sahara Desert from the subtropical regions of Africa (***Figures 1*** and ***2***). The Sahel encompasses significant portions of Mauritania, Mali, Niger, Chad, Sudan, and Eritrea and touches smaller portions of other nations, including Nigeria. The Sahel is characterized by a short, increasingly unpredictable rainy season that punctuates an otherwise arid landscape. While droughts are typical of the region, climate change has resulted in more consistent and severe drought events [6,7]. The frequent disappearance of the rainy season makes the area one of the most vulnerable to climate change-related impacts [8]. Traditional lifestyles such as farming and raising livestock have become increasingly difficult and fueled long-standing tensions between agrarian and pastoralist tribes. Because the Sahel is not only characterized by ecoclimatic conditions, but also by political instability, poverty, urbanization, and conflict over resources, the region has been referred to as “ground zero” for climate-related conflict [6]. In assessing areas in Africa with the greatest exposures to climate-related hazards, Busby et al. found that the Sahel repeatedly displayed higher vulnerabilities, and northern Nigeria ranked among the highest exposure quintiles [8].

Similar to the Sahel, lack of adequate rainfall and limited resources are straining already tense relations between Zamfara’s pastoralist (Fulani) and agrarian (Hausa) tribes [9,10,11]. The instability that characterizes much of the Sahel region is playing out in the “forgotten crisis” in Zamfara State [12]. Examining the complex relationships between conflict, climate change, and resource extraction in this region provides an opportunity to apply lessons learned to vulnerable regions beyond Nigeria.

## Zamfara Environmental Health Crisis

### Zamfara State’s Short and Turbulent History

The area that is now Zamfara was part of Nigeria’s Sokoto State until 1996, when the *Zamfara-wa* ethnic group of the larger Hausa tribe succeeded in their demand for autonomy [13]. That same year in nearby Kano State, the pharmaceutical company Pfizer Inc. launched an experimental meningitis treatment trial. At best a hastily designed trial and at worst an ethical abomination, of the 200 children given the experimental drug, five died and dozens were left with permanent disabilities [14,15]. Imams broadcasted warnings on the radio, accusing the US company of attempting to kill or sterilize Muslim children and advising parents against seeking any form of western medicine. Polarization and distrust between tribes and religious groups flared, and vaccination rates plummeted [16]. Allegations against Pfizer were publicized in 2000 [15], the same year Sharia Law was implemented in Zamfara. A lawsuit filed in the US against Pfizer was dismissed, but a lawsuit later filed in Nigeria resulted in at least 4 families receiving compensation for their claims against the company [17].

Rates of preventable diseases soared, and polio made a resurgence [16,18]. Aid organizations such as Médecins Sans Frontières (MSF, Doctors Without Borders) began regularly surveilling for outbreaks of cholera, malaria, measles, and meningitis. MSF was on such a surveillance mission in 2010 when health workers in remote areas of Zamfara reported high mortality rates in children in Anka and Bukkuyum Local Government Areas [1]. Acute lead poisoning was eventually identified as the cause, and a multidisciplinary response team investigated and found the affected communities were deeply involved in artisanal gold mining [2].

### Mass Childhood Lead Poisoning Crisis and Response

**Figure 3:**
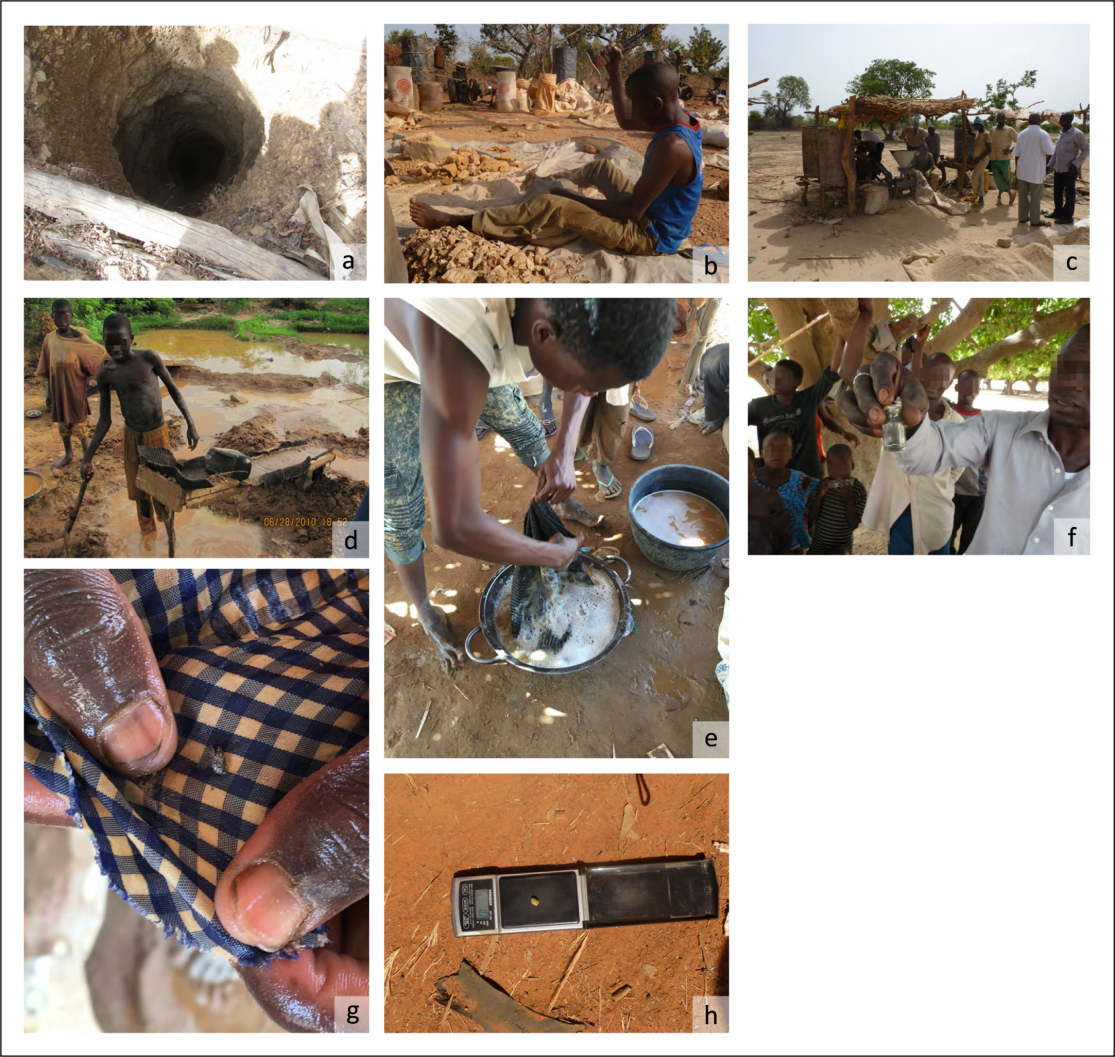
Photo series of gold ore processing in Zamfara State, Nigeria: **a**, ore is sourced from mines across the region and transported to processing areas; **b**, ore rock is broken by hand into gravel-size pieces. **c**, modified grain mills are used to grind ore into a flour-like consistency; **d**, sluice boards are often used to gravitationally separate gold particles from silica and other contents; **e**, contents of the sluice carpets are washed into a container; **f**, mercury is added to the sluiced concentrate; **g**, a mercury-gold amalgam is obtained, which will then be heated, vaporizing the mercury; **h**, the final product is ready to be sold to dealers who visit the mine sites regularly. Photo credits: TIFO, Casey Bartrem, Simba Tirima, and Ian von Lindern.

As in many areas of northern Nigeria, and in an increasing number of African countries, people have turned to artisanal and small-scale mining (ASM) as a major source of economic security [9]. Due to the wide variety in mining processes, organizational structures, and mineral commodities, definitions of ASM vary, but it generally encompasses mining operations without advanced technology and varying organizational structures [19]. One of the most common and rapidly growing forms of ASM is artisanal and small-scale gold mining (ASGM) (***Figure 3***).

Lead poisoning associated with ASGM is rare; typically, it is the use of mercury in isolating gold from impurities in ores that is the focus of human health risk assessments. The severe mortality and morbidity in Zamfara have three principle causes. First, small-scale mining efforts among impoverished Hausa villages rapidly increased when gold prices shot up during the 2008 economic crisis. Second, a new vein of ore was discovered late in 2009 that, in a cruel twist of geologic fate, contained 10% (100,000 mg/kg) lead [20]. And third, ore was processed in residential areas, either in public areas around the community or within the walled homes by women secluded under the Sharia Law practice of *purdah*.

**Figure 4:**
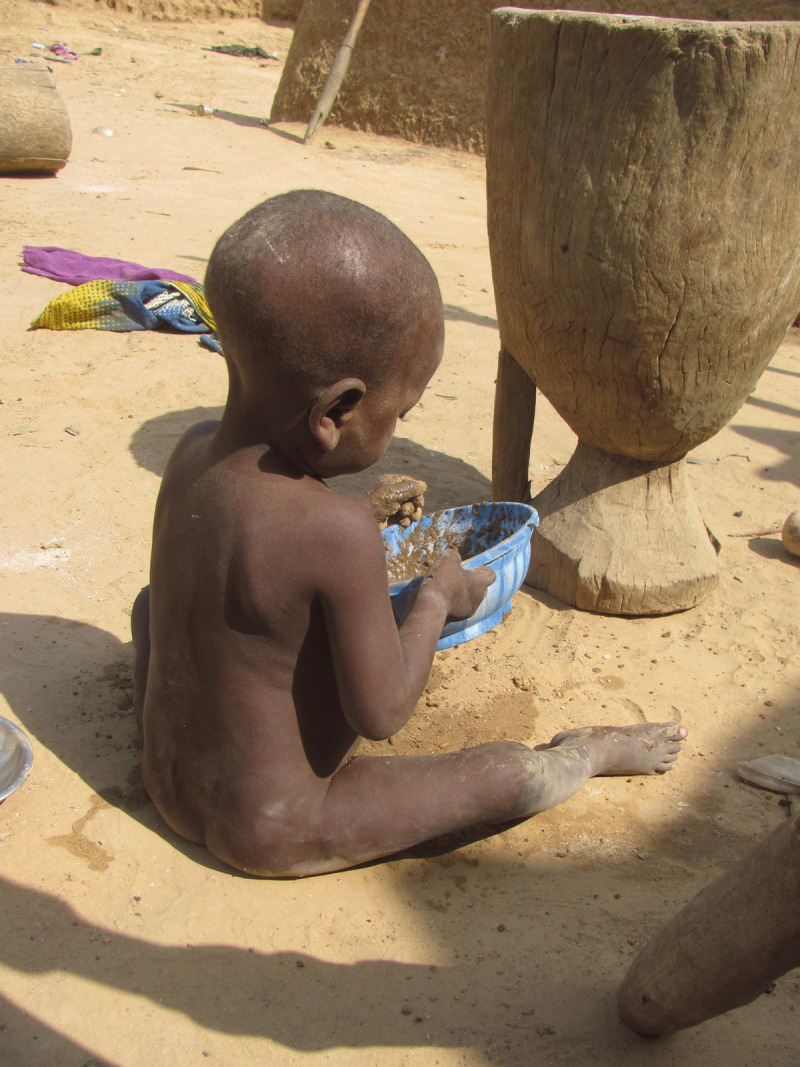
A boy plays with soil on the floor of his home (photo credit: Casey Bartrem Casey Bartrem/TIFO).

**Figure 5:**
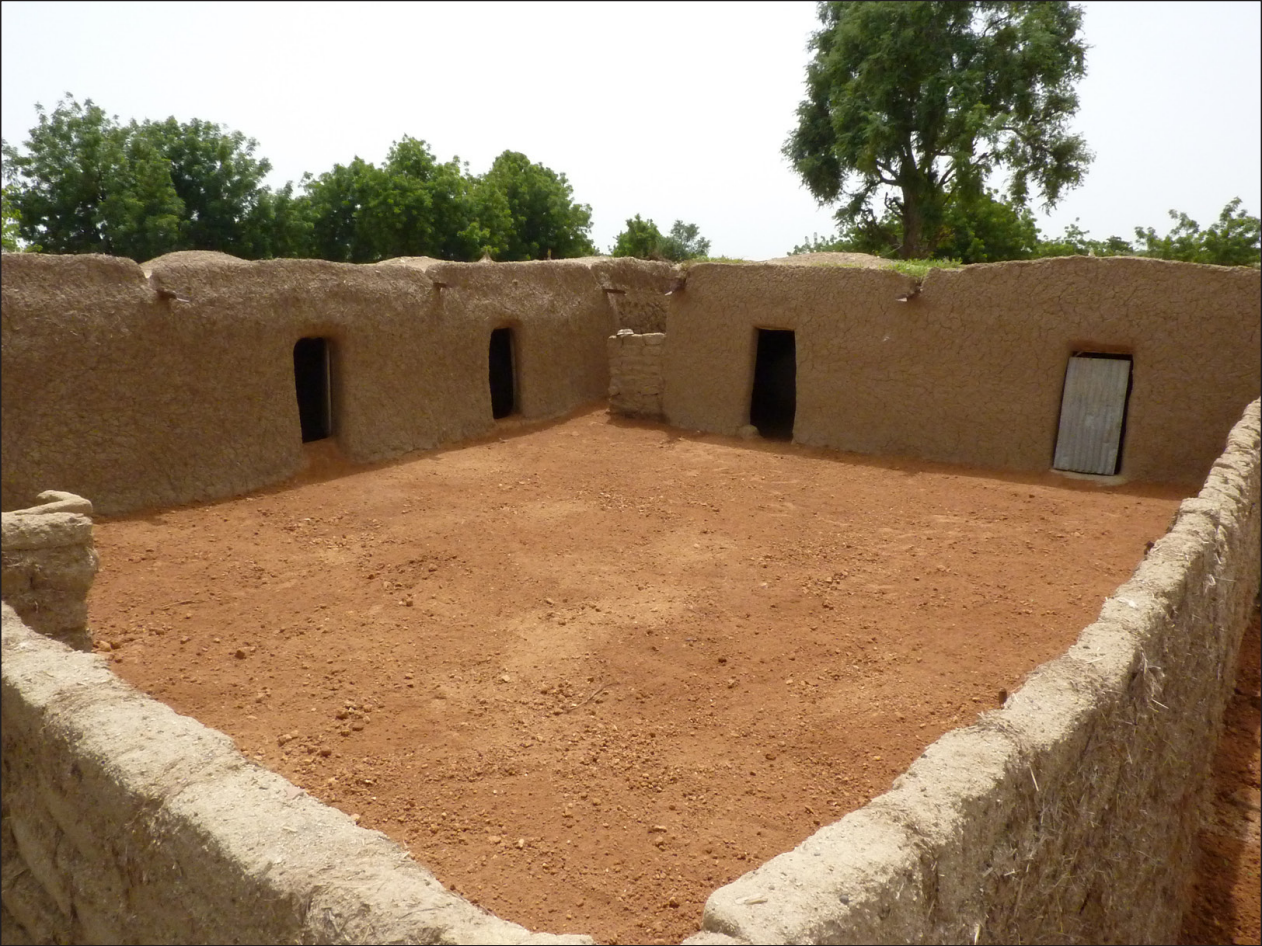
A recently remediated home in Zamfara State. Contaminated surface soils have been excavated and replaced with soils certified to be <20 mg/kg Pb (photo credit: Casey Bartrem Casey Bartrem/TIFO).

Lead is a potent neurotoxin that is especially harmful to the developing brains of young children. In Zamfara in 2009–10, lead-rich ore dust covered the soil floors of homes (***Figures 4*** and ***5***), became incorporated into foods prepared and served on the ground, and rapidly left many children encephalopathic, leading to seizures, coma, and death [3,21,22]. Soil lead levels exceeded the USEPA residential soil limit of 400 mg/kg by orders of magnitude, reaching up to 100,000 mg/kg (10%) in multiple homes [23,24]. Geometric mean blood lead levels (BLLs) were 149 µg/dL in 2010, nearly 30 times the CDC 5 µg/dL level of concern, with maximum BLLs over 700 µg/dL [1,25]. Up to 1 of every 3 young children died in the most severely impacted villages [2,26,27]. More than 17,000 people were exposed to excessively high concentrations of lead in the environment [1,28].

Over the next three years, TerraGraphics International Foundation (TIFO) worked with MSF and the Zamfara State and Nigerian federal governments to address the epidemic in eight Zamfara communities. The response was unprecedented in both Nigeria and the world. Cleanup procedures were modified to accommodate emergency medical treatment timelines, limited resources, remote location, and Sharia Law [1,24]. Mortality rates decreased from 30% to <2% [1]. The last Zamfara village was remediated in 2013; two years later, a similar ASGM-related lead poisoning outbreak was discovered in neighboring Niger State [29]. The Nigerian government, MSF, and TIFO collaborated to respond again, addressing exposures in two remote villages. MSF has continued to treat children for lead poisoning with chelation therapy in the impacted villages, expanding the medical program to include support for local institutions to manage transient recontamination that occurs from mining camps. MSF also supports a safer mining program to reduce lead and silica dust exposures for workers in the region [30,31].

### Humanitarian Crisis in Zamfara

**Figure 6:**
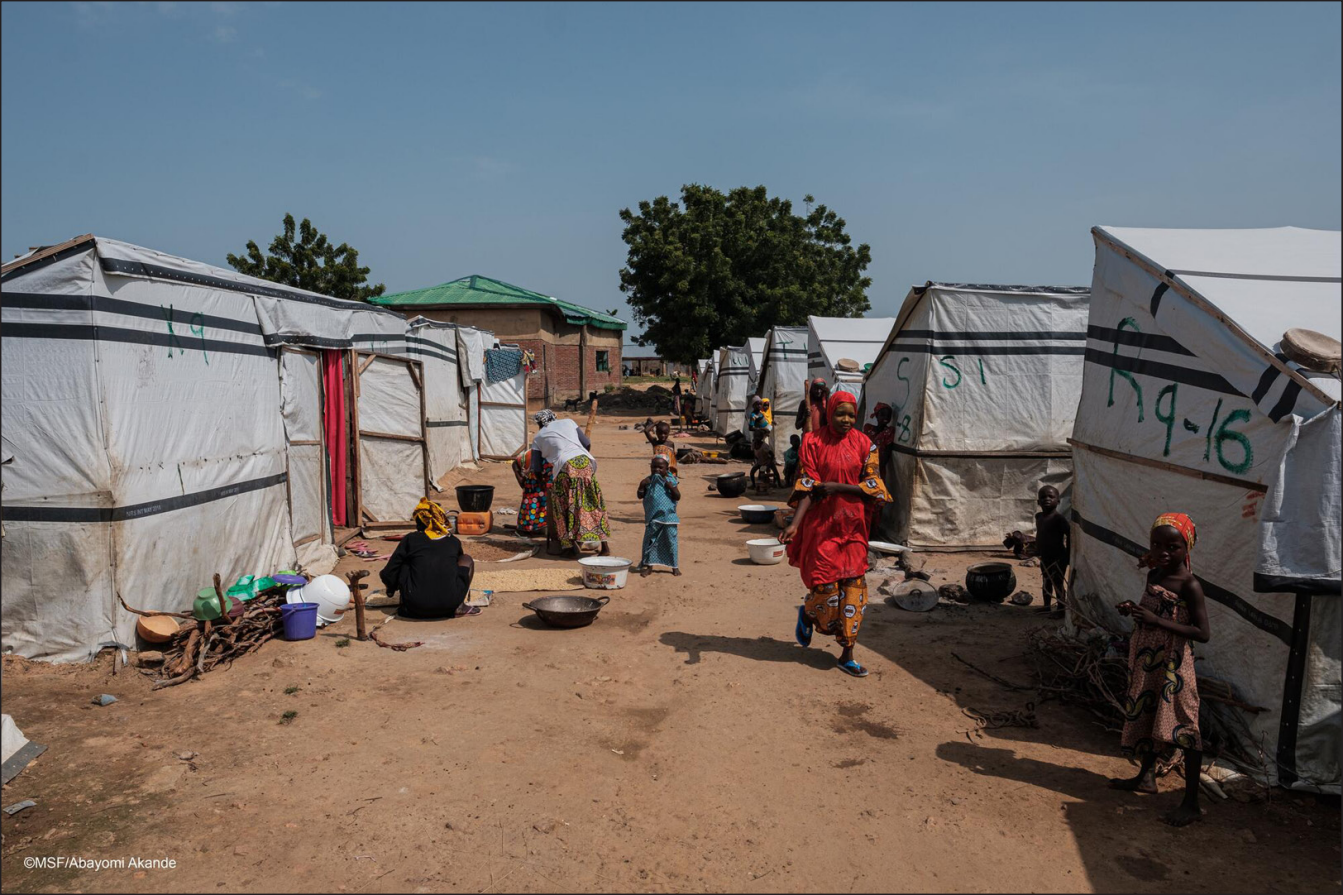
A camp for people displaced by regional violence in Anka, Zamfara State, Nigeria in October 2020. Many people living in the camp were once residents of the villages impacted by lead poisoning in the previous decade (photo credit: MSF/Abayomi Akande [45]).

The context has changed dramatically since 2010 when the greatest security threat in northern Nigeria was Boko Haram’s terrorism of communities in Borno State in the Lake Chad region. Today, clashes between ethnic groups—principally the agrarian Hausa tribe and the pastoralist Fulani Tribe—over land use in Zamfara have resulted in more deaths and displacement than terrorism in Borno [11,32,33]. In Zamfara in 2018, 21,000 people had fled their homes, 10,000 people had died, and 44,000 children had been orphaned [9,34,35]; as of January 2021, the number of IDP from Zamfara had increased to 70,000 [36] (***Figure 6***). Despite military presence, vigilante groups patrol the area, kidnappings are rampant, and many rural villagers have fled, fearing for their lives [5,37,38].

The deteriorating security has complex roots in long-standing ethnic tensions, inadequate governance, and extreme poverty, similar to the situation in 2009 when Boko Haram’s terrorism of Borno began [10,35,39]. Just as each of these factors alone cannot explain the acute crisis in Zamfara, analysis of the underlying causes without consideration of how climate change has exacerbated the issues would be neglecting a crucial part of the story. Desertification in the Sahel and northern reaches of Nigeria has pushed herders south, where existing tensions over land have resulted in conflicts between pastoralist and agrarian tribes for centuries [9,40]. Competition for agricultural land versus grazing land, and access to regions with adequate water for both, continues to fuel the Zamfara conflict [35,41].

The security situation has serious implications for MSF’s lead chelation treatment program. Medical teams can no longer access impacted villages to support safer mining practices or to distribute medications due to the high risk of robbery or kidnappings on the roads [5,42]. The organization currently runs a camp in a nearby town for people displaced by the violence [43]. In response to the violence, the Nigerian federal government enacted a temporary ban on all artisanal gold mining, but many experts doubt that this will have an impact on the security situation and fear that it could force the industry “underground” and recreate unsafe ore extraction activities that caused the high mortality seen in 2010 [44].

Where ASGM had the potential to bring economic opportunity and stability to an otherwise severely impoverished region, the current situation poses risks for Zamfara, Nigeria, and the entire region. The increase in tensions and interruption of income generating activity could easily result in a rise of extremism in Zamfara, a region where it has historically been absent [12]. Meanwhile, extremist activity is on the rise in nearby Burkina Faso, where thousands of IDP are fleeing violence and ASGM areas are now being targeted by jihadi groups [46].

**Figure 7:**
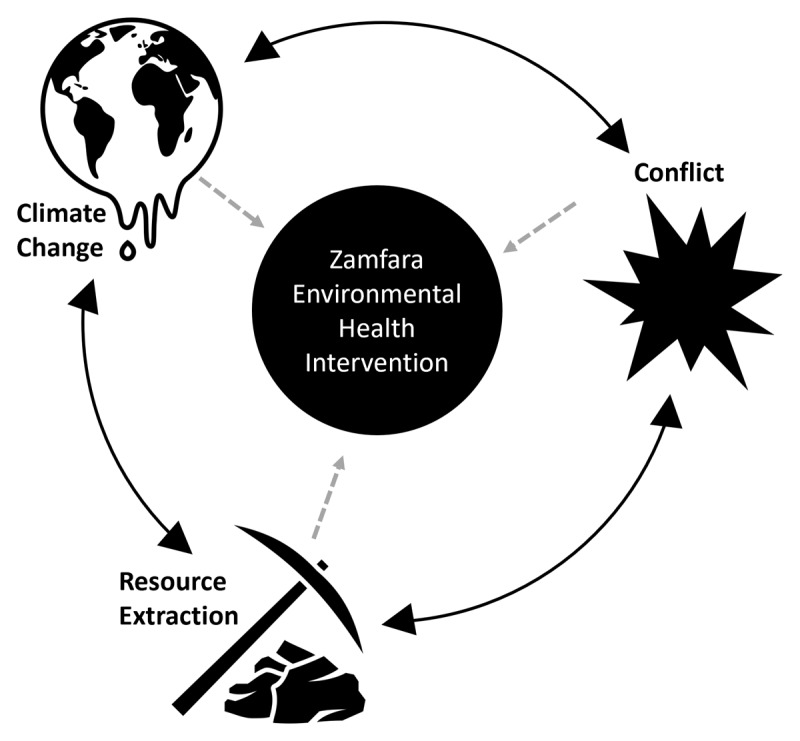
Climate change, conflict, and resource extraction, as well as the interactions between these issues, are resulting in significant impacts on the environmental health project in Zamfara State, Nigeria.

The challenges Zamfara now faces—climate change, managing a booming ASGM economy, and escalating conflict—are not unique. Many governments face the daunting task of managing the same challenges. Estimates vary, but there up to 100 million people who rely on ASGM today [47]. While climate change impacts are global, certain regions of the world are known to be disproportionately impacted [6]. Countries experiencing these issues may also be dealing with other public health crises, political unrest, severe poverty, and environmental degradation [35,48,49]. This greatly complicates governance, adaptation and mitigation strategies, emergency interventions, humanitarian actions, and development initiatives. There is mounting evidence that conflict, climate change, and resource extraction are not only related, but fuel each other (***Figure 7***) [7,50,51]. This is a wicked problem because of the intractable set of interdependencies, lack of clear solution(s), and the potential for creating additional challenges when attempting to solve one aspect of the problem [52].

## Climate Change and Conflict

Domestic and international security experts recognize climate change as a critical national security concern. The increased likelihood of interstate conflict as well as violent conflicts within countries intensifies the need for both domestic and international humanitarian aid [7]. Experts first suggested a plausible link between global climate change and multiple forms of conflict in 2007 [53,54,55]. In the US, climate change is acknowledged as a crucial consideration for infrastructure protection, environmental degradation, public health, and conflict [56]. In 2010, the US Department of Defense stated “While climate change alone does not cause conflict, it may act as an accelerant of instability and conflict… [7]” United Nations (UN) Secretary-General Ban Ki-moon stated that “Climate change not only exacerbates threats to international peace and security; it is a threat to international peace and security [57].”

While the concept of climate-induced human conflict is not new, it has been contested [58,59,60,61]. One issue in validating the link between climate change and conflict is the complexity of climate-related changes that can fuel human conflicts. Devlin and Hendrix found that precipitation variability had a greater likelihood of resulting in conflict than overall water scarcity [62]. And yet, Raleigh et al. analyzed market food prices and the occurrence of violent conflict and found not only do elevated food costs positively correlate to risk of conflict, dry conditions are a likely culprit for the relationship [63]. A significant confounding issue is that proponents of the climate change-conflict theory have not been able to fully demonstrate mechanisms by which the effects occur [62,64].

Despite the challenges in determining the causal relationships, reviews and meta-analyses of available literature indicate that climate-related issues are significant contributors to various types of conflict, with an abundance of articles to support the theory [64]. For example, studies investigating historical relationships between climate and conflict have found consistent patterns. Zhang et al. found climate change to be a driver of armed conflict during a 300 year period in North America [55]. The same lead author also demonstrated a correlation between war in eastern China and temperature changes that altered food production over the course of 1000 years [54]. Analyses of more recent climate-related security issues are also compelling. Busby et al. found evidence for conflicts related to flooding, droughts, and population density [65]. They also note the exacerbation of pre-existing vulnerabilities and challenges in governments’ capacity to respond to issues, ranging from inadequate infrastructure to violence [65]. In countries affected by El Nino events between 1950–2004, the chance of civil war doubled during warmer periods [66]. Other authors have amassed significant evidence for climate change-related conflict due to temperature changes, altered rainfall patterns, and sea level rise [64]. Such findings are in line with the occurrence of resource-related conflicts that occur in both renewable and nonrenewable resources globally [67].

## Conflict and Resource Extraction

In 1993, Richard Auty used the term “resource curse” in reference to the paradoxical relationship between a country’s high-value natural resources and that country’s low social, economic, health, and environmental development indicators [50]. Because resource extraction, unlike other sectors, can occur without the development of domestic, political, or economic governance, conflicts are more likely to occur over these commodities [68]. These conflicts may be large-scale (e.g., civil war) or small-scale (e.g., among individuals or groups) [6]. From 1970 to 2008, roughly half of global armed conflicts were related to high-value natural resources [69]. In Africa, three of the most violent wars in recent years were related to extractive industries [70]. More recently, ASM has been linked to violent conflict in multiple countries, including Columbia, Democratic Republic of the Congo (DRC), and in the Sahel [46,71].

ASM can also stabilize a region when well-managed and supported [72,73]. In Sierra Leone, diamond mining transitioned from fueling violent civil war to being instrumental in peacebuilding by reducing extreme poverty and boosting government revenues to fund social programs [74]. Responsible, bottom-up strategies for formalizing, supporting, and regulating ASM have proven economic and environmental benefits when governance puts human health and welfare as the policy focus [75,76,77,78,79]. ASM has been proposed as both a development strategy and a climate change adaptation strategy [75,79]. Yet policies that promote healthy ASM communities and generate revenue to support sustainable practices are scarce [80]. In 2016 alone, the United Arab Emirates reported over 15 billion USD in gold imported from Africa, a 10-fold increase from 2006. More than 10 billion USD of this import went unrecorded by African nations, highlighting not only a massive loss in potential revenue, but a missed opportunity to fund health initiatives for mining communities [81].

While there are clear associations between ASM, conflict, and adverse environmental health outcomes, banning the activity has consistently proven to be both ineffectual and counterproductive [75,77,78,82]. ASM is a poverty-driven activity; outright bans negatively impact ASM communities by disrupting or completely severing sources of income, further destabilizing communities and regions [73,75,76,83,84]. Responsible regulation that goes beyond simply taxing miners is a more effective way to reduce adverse impacts of ASM [77]. These support strategies are best developed at the local level and include miners, community leaders, vulnerable groups, and multiple levels of formal leadership and governance [76,85,86]. Such strategies will be more effective at curbing negative impacts, and will also improve overall programmatic sustainability.

## Resource Extraction and Climate Change

Both informal and formal mining industries impact and are impacted by climate change. Mine operations, and associated deforestation, are contributors to greenhouse gas emissions [87,88]. Climate change impacts associated with mineral extraction and production doubled between 2000 and 2015 [89]. But large scale mining companies are also evaluating climate change operational impacts and implementing adaptation strategies related to infrastructure improvements, water scarcity predictions, and special considerations in northern climates where permafrost is melting [87,90,91,92,93]. In 2017, El Salvador named climate change concerns as a major reason in the decision to ban all mineral mining in the country, citing concerns over water scarcity and increasing threats to tailings dams from extreme weather events [93].

The evidence of a link between climate change and informal sector mining is more limited. Nonetheless, the potential relationship is important due to the extensive occurrence and explosive growth of the sector. ASM is often referred to as informal, but ranges from the individual level to highly organized systems, and is found throughout mineral-rich regions of Africa, the Americas, and Asia [94]. As of 2017, an estimated 40 million people worked in ASM globally, compared to 7 million employed in industrial-scale mining [80]. It is estimated that 15–20% of global minerals, including 80% of sapphires and 30% of gold, are produced by small-scale artisanal miners [95]. An estimated 14–19 million people globally are directly employed by ASGM alone [48,96], but the economy supports more than 100 million people, ten times more than before the 2008 world economic crisis [47]. In some countries, ASM production exceeds industrial mining. For example, in the DRC, more than 50% of coltan (an ore containing tantalum, a component used widely in electronics) and more than 90% of gold are informally produced [77].

While ASM is largely fueled by commodity prices and lack of viable economic alternatives [97], other environmental factors also contribute to shifts in this informal sector work. Some authors suggest that environmental degradation has contributed to the growing artisanal mining economy [75,93,94]. In the face of decreasing crop yields, unpredictable precipitation, droughts, and increasing demands on grazing and agricultural lands, both agrarian and pastoralist groups are increasingly unable to pursue traditional livelihoods [9,75,98]. In rural areas, mining represents an important means of income diversification for many farmers, and addressing both agriculture and mining issues is necessary for lasting impacts on development [51,99]. In areas with distinct dry and rainy seasons, ASM allows farmers to generate income during the dry seasons while farming during the rainy season. Due to declining value of many export crops, loss of agriculture input subsidies (e.g., fertilizers), and changing weather patterns (rainfall), off-season workers increasingly rely on ASM [51]. Despite these relationships, there is limited research on the potential impacts of climate change on the growing ASM economy [93].

Climate change will fuel both the formal and informal mining sectors in another important manner. Demand for alternative energy options is expected to significantly alter the global mineral economy [89,100]. Demand for cobalt, copper, and rare earth elements used in batteries, electric vehicles, wind turbines, and other “renewable” energy components will increase by several orders of magnitude [100]. This suggests that such low-carbon alternatives should not be referred to as “green” or “renewable” technologies given the environmental, social, and health impacts associated with sourcing their components, which are finite resources.

## Additional Considerations and Recommendations

During a national conference on lead poisoning associated with ASGM in Abuja in 2018, the Nigerian Vice President stated “As Nigeria traverses the road to shared mining prosperity, we must ensure that we do not do it in a way that harms our health or environment. Those who say the option is death by poisoning, rather than poverty, offer a cynical choice [101].” The worrisome destabilization of Zamfara since the 2018 conference serves to highlight the importance of supporting healthy ASM communities in Nigeria. Accomplishing this requires attention to several key issues.

First, assessing and addressing environmental health challenges associated with ASM cannot focus on a single risk. The most often cited environmental health risk related to ASGM is from the use of elemental mercury in isolating gold particles. Mercury is a highly toxic metal that impacts the brain and kidneys of workers and communities [96,102], and it is also a global pollutant distributed via atmospheric and hydrologic pathways [103,104,105]. ASGM now is the largest source of global mercury pollution [106,107]. However, focusing solely on mercury in ASGM neglects other substantial occupational and environmental health risks. ASM workers experience exposures to silica dust, multiple heavy metals, and cyanide, musculoskeletal injuries, hearing damage, and mine collapses [48,104]. Mining communities face increased risk of water and vector borne diseases, higher rates of sexually transmitted diseases, and alcohol and drug abuse [48,108]. Many ASM-related health crises place burdens on governments or humanitarian actors to respond to violence or disease outbreaks [49]. Comprehensive strategies must be developed in consideration of all of these risks.

In assessing and mitigating the impacts of and interactions between climate change, conflict, and resource extraction, special consideration must be given to vulnerable groups. Women experience more severe climate change impacts due to financial inequalities, barriers to property ownership, and neglectful or discriminatory agricultural extension programming resulting in limited access to improved seed varieties, land credit, and fertilizers, all of which are essential in managing climate-related challenges [109]. Children are more vulnerable to malnutrition resulting from climate-related decreases in crop yields, increased rates of vector-borne diseases, and increased levels of air pollution [110]. Female miners are also marginalized or neglected from ASM formalization efforts [111]. During conflict, women are subject to sexual assault, psychological abuse, and lack of access to reproductive care, and they experience higher maternal mortality rates [112,113]. At mining sites, women often experience violence, abuse, and discrimination, and pregnant women are more vulnerable to chemical and physical hazards [114,115]. Children often suffer from abuses related to child labor [104,116] and are particularly sensitive to chemical exposures during sensitive periods of neurodevelopment. Toxicants such as mercury and lead can pass the blood-brain and placental barriers, impacting critical neurodevelopment and resulting in impacts ranging from IQ deficits to severe brain damage [102,117,118].

Some of the direct adverse outcomes associated with ASM are addressed by industrial-scale mining operations. Proponents of formalization point to better occupational health and safety practices, banning of child labor, and improved job security for workers. There is some merit to these claims, but there are significant economic, environmental, and social costs to these types of formalized mining operations. The inherent nature of formal mining includes efficiencies related to mechanization rather than human-powered operations. Hence, the economic benefits are limited to a smaller number of mostly male miners, often resulting in mining communities where few people benefit monetarily [111,119]. Yet everyone in these communities suffers from the environmental degradation associated with the operations [120]. Further, large-scale operations tend to be foreign-owned, further reducing the distribution of wealth. Formalization should therefore focus on legalization and support of equitable ASM, not on shifting ASM practices towards industrial-scale mining.

In all of these efforts, any viable solution will be interdisciplinary, and any sustainable solution will be locally implemented. In Zamfara, the environmental health response was modeled on an approach similar to that used at a US Superfund Site [1]. Those validated scientific methods were adapted and implemented by local institutions. This is crucial for all environmental health interventions but is highlighted by the subsequent humanitarian crisis in Zamfara. When international aid organizations are no longer able to regularly access impacted communities, it is local authorities who will be responsible for managing ASGM in a way that prevents future health crises.

While understanding the geo-political realities in a region is important, hundreds of frameworks have been developed for analyzing relationships between health and climate change, climate change and the environment, and even mining and climate change, yet none capture the full scope of the interactions between all the challenges. Further, existing models are not appropriate for situations where the dominant extractive industry is in the informal sector, a growing reality in many countries [70,93,121]. The climate change, conflict, mining nexus is a wicked problem because it is inherently interdisciplinary, has complex interdependencies, crosses international borders, is geographically and temporally dynamic, and is difficult to both define and solve [56]. In the face of these challenges, involvement from local stakeholders in designing site-specific adaptation and intervention strategies is paramount.

It is encouraging to see more recent research into the impacts of climate change on formal sector mining, but the relationship between climate change and informal mining needs further investigation. The interactions between climate change and ASM include direct effects, such as people giving up increasingly tenuous subsistence lifestyles for more reliable income and indirect effects, including the shift in mineral demand due to increasing use of alternative energy options.

## Conclusions

Environmental health crises related to ASM will continue to occur in marginalized communities for as long as global markets demand coltan, gold, and other minerals. This was clearly demonstrated in Zamfara, where the dramatic increase in gold value was partially responsible for the lead poisoning outbreak [1,3,24]. Demand for metals is expected to intensify with the production of low-carbon energy alternatives. The World Bank estimates a 585% increase in the demand for cobalt by 2050 in order to meet demand for renewable energy alone [122]. Most of the world’s cobalt comes from mining in the DRC, a country that has been embroiled in violent conflict for decades and where other mineral resources, such as coltan, are used to fund multiple armed groups [123,124]. With increased global demand for and production of mineral resources, there is a risk that conflicts over nonrenewable resources will amplify if environmental and health impacts are not mitigated and economic benefits are not equitably distributed [9,67,125].

**Figure 8:**
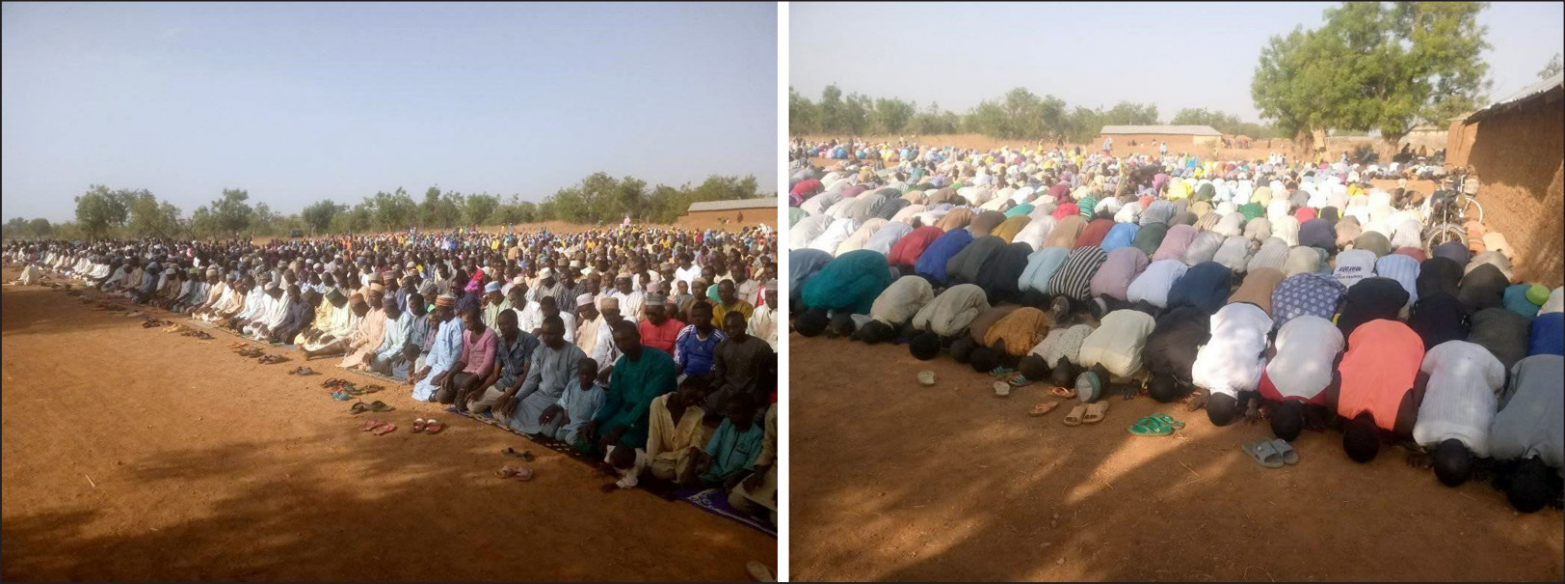
Residents of Bagega Village in Zamfara State, Nigeria at a mass prayer for peace (2020, photo credit: Simba Tirima).

A combination of explosive population growth, pervasive climate change impacts, and vast quantities of unexploited mineral resources place the African continent in an especially precarious position in coming decades [126,127]. By the end of this century, Africa will be home to half of the world’s children and 40% of the global population [128]. Nigeria is one of nine countries where the largest increase in population is expected to occur and it is no stranger to conflict: the Niger Delta region has been plagued with intractable conflict related to oil and gas extraction for decades [129,130,131]; the devastating Biafra conflict in the late 1960s was the impetus for several French physicians to form what is now MSF [132]; and the unethical Pfizer drug trial and its effects are still apparent in today’s polio survivors [14,15]. Zamfara exemplifies a region grappling with managing the challenges of conflict (***Figure 8***), climate change, and resource extraction against a regional backdrop of extreme poverty, a rapidly increasing population, health crises, and environmental degradation. Locally driven, sustainable climate change adaptation strategies inclusive of responsible ASM could be a foundation for stabilizing impacted communities and promoting peace.

## References

[1] Tirima S, Bartrem C, von Lindern I, et al. Environmental remediation to address childhood lead poisoning epidemic due to artisanal gold mining in Zamfara, Nigeria. Environ Health Perspect. 2016; 124(9). DOI: 10.1289/ehp.1510145. PMC5010416 26745642

[2] Dooyema CA, Neri A, Lo YC, et al. Outbreak of fatal childhood lead poisoning related to artisanal gold mining in Northwestern Nigeria, 2010. Environ Health Perspect. Published online December 20, 2011. DOI: 10.1289/ehp.1103965 PMC3339453 22186192

[3] von Lindern I, von Braun M, Tirima S, Bartrem C. Zamfara, Nigeria lead poisoning epidemic emergency environmental response final report. TerraGraphics Environmental Engineering, Inc.; 2011. Accessed September 22, 2016. http://www.terragraphicsinternational.org/publications.

[4] Thurtle N, Greig J, Cooney L, et al. Description of 3,180 courses of chelation with dimercaptosuccinic acid in children ≤5 y with severe lead poisoning in Zamfara, Northern Nigeria: A retrospective analysis of programme data. PLOS Med. 2014; 11(10): e1001739. DOI: 10.1371/journal.pmed.1001739. 25291378 PMC4188566

[5] MSF. On the run from violence in Zamfara state. Médecins Sans Frontières (MSF) International. Published October 15, 2019. Accessed December 12, 2019. https://www.msf.org/run-violence-nigeria.

[6] UNEP. Livelihood security: Climate change, migration and conflict in the Sahel. United Nations Environment Programme; 2011. Accessed July 21, 2019. https://postconflict.unep.ch/publications/UNEP_Sahel_EN.pdf.

[7] National Research Council. Climate change as a national security concern. In: Climate and Social Stress: Implications for Security Analysis. The National Academic Press; 2013. DOI: 10.17226/14682

[8] Busby JW, Cook KH, Vizy EK, Smith TG, Bekalo M. Identifying hot spots of security vulnerability associated with climate change in Africa. Clim Change. 2014; 124(4): 717–731. DOI: 10.1007/s10584-014-1142-z

[9] Suleiman S. Rural banditry in Zamfara state, Northwest Nigeria. Kujenga Amani. Published June 13, 2019. Accessed July 3, 2019. https://kujenga-amani.ssrc.org/2019/06/13/rural-banditry-in-zamfara-state-northwest-nigeria/.

[10] Anyadike O. Zamfara: Nigeria’s wild northwest. New Humanit. Published online September 13, 2018. Accessed July 3, 2019. https://www.thenewhumanitarian.org/news-feature/2018/09/13/zamfara-nigeria-s-wild-northwest.

[11] Amnesty International. Nigeria: Thousands living in fear as Zamfara armed bandits ramp up attacks. Published July 31, 2018. Accessed July 3, 2019. https://www.amnesty.org/en/latest/news/2018/07/nigeria-thousands-living-in-fear-as-zamfara-armed-bandits-ramp-up-attacks/.

[12] Ahmed K. Letter from Africa: Why is no-one talking about the Zamfara conflict? BBC News. https://www.bbc.com/news/world-africa-44126529. Published May 18, 2018. Accessed July 8, 2021.

[13] Zamfara State. Zamfara State Government – Official website for the State. Accessed July 8, 2021. https://zamfara.gov.ng/.

[14] Ahmad K. Drug company sued over research trial in Nigeria. The Lancet. 2001; 358(9284): 815. DOI: 10.1016/S0140-6736(01)06011-1 11564494

[15] Stephens J. Where profits and lives hang in balance. Washington Post. https://www.washingtonpost.com/archive/politics/2000/12/17/where-profits-and-lives-hang-in-balance/90b0c003-99ed-4fed-bb22-4944c1a98443/. Published December 17, 2000. Accessed June 23, 2019.

[16] Jegede AS. What led to the Nigerian boycott of the polio vaccination campaign? PLOS Med. 2007; 4(3):e73. DOI: 10.1371/journal.pmed.0040073 17388657 PMC1831725

[17] The Guardian. Pfizer pays out to Nigerian families of meningitis drug trial victims. The Guardian. Published August 12, 2011. Accessed July 8, 2021. http://www.theguardian.com/world/2011/aug/11/pfizer-nigeria-meningitis-drug-compensation.

[18] Simpson S. Government blames polio vaccine boycott on Pfizer trials. New Humanit. Published online June 7, 2007. Accessed July 3, 2019. http://www.thenewhumanitarian.org/report/72601/nigeria-government-blames-polio-vaccine-boycott-pfizer-trials.

[19] Burki TK. The true scale of artisanal mining. Lancet Respir Med. 2019; 7(5): 384–385. DOI: 10.1016/S2213-2600(19)30115-8 30962166

[20] Plumlee GS, Durant JT, Morman SA, et al. Linking geological and health sciences to assess childhood lead poisoning from artisanal gold mining in Nigeria. Environ Health Perspect. 2013; 121(6): 744–750. DOI: 10.1289/ehp.1206051 23524139 PMC3672918

[21] Tirima S, Bartrem C, von Lindern I, et al. Food contamination as a pathway for lead exposure in children during the 2010–2013 lead poisoning epidemic in Zamfara, Nigeria. J Environ Sci China. 2018; 67: 260–272. DOI: 10.1016/j.jes.2017.09.007 29778160

[22] Greig J, Thurtle N, Cooney L, et al. Association of blood lead level with neurological features in 972 children affected by an acute severe lead poisoning outbreak in Zamfara State, northern Nigeria. PloS One. 2014; 9(4):e93716. DOI: 10.1371/journal.pone.0093716 24740291 PMC3989233

[23] USEPA. Federal Register: Environmental Protection Agency, 40 CFR part 745, Lead; Identification of dangerous levels of lead; final rule. Published online January 5, 2001. http://www.epa.gov/fedrgstr/EPA-TOX/2001/January/Day-05/t84.pdf.

[24] Bartrem C, Tirima S, von Lindern I, et al. Unknown risk: co-exposure to lead and other heavy metals among children living in small-scale mining communities in Zamfara State, Nigeria. Int J Environ Health Res. 2014; 24(4): 304–319. DOI: 10.1080/09603123.2013.835028 24044870

[25] CDC. Lead levels in children. Childhood lead poisoning prevention. Published July 31, 2019. Accessed December 12, 2019. https://www.cdc.gov/nceh/lead/prevention/blood-lead-levels.htm.

[26] Lo YC, Dooyema CA, Neri A, et al. Childhood lead poisoning associated with gold ore processing: a village-level investigation—Zamfara State, Nigeria, October–November 2010. Environ Health Perspect. 2012; 120(10): 1450–1455. DOI: 10.1289/ehp.1104793 22766030 PMC3491928

[27] Biya O, Gidado SO, Haladu S, et al. Notes from the field: Outbreak of acute lead poisoning among children aged <5 years — Zamfara, Nigeria, 2010. Centers for Disease Control and Prevention; 2010: 846–846. http://www.cdc.gov/mmwr/preview/mmwrhtml/mm5927a3.htm?s_cid=mm5927a3_e%0D%0A.

[28] WHO. Nigeria: mass lead poisoning from mining activities, Zamfara State – Update 1. Published November 11, 2011. http://www.who.int/csr/don/2011_11_11/en/.

[29] Sahara Reporters. 28 children die by lead poisoning In Niger State from gold mines. Sahara Reporters. Published May 15, 2015. Accessed July 3, 2019. http://saharareporters.com/2015/05/15/28-children-die-lead-poisoning-niger-state-gold-mines.

[30] Gottesfeld P, Tirima S, Anka SM, Fotso A, Nota MM. Reducing lead and silica dust exposures in small-scale mining in Northern Nigeria. Ann Work Expo Health. 2019; 63(1): 1–8. DOI: 10.1093/annweh/wxy095 30535234

[31] MSF. Nigeria. Medecins Sans Frontieres. Accessed August 13, 2021. https://www.msf.org/nigeria.

[32] Sunday O. This Nigerian conflict Is proving deadlier than Boko Haram. OZY. Published online May 5, 2019. Accessed July 3, 2019. http://www.ozy.com/fast-forward/the-little-known-nigerian-conflict-thats-more-deadly-than-boko-haram/94040.

[33] Yahaya JU, Bello MM. The rise of banditry and its attendant effect in governance and socio-economic relations in Zamfara State. Polca Int J Humanit Secur Stud. 2020; 5(1): 109–129.

[34] Akinwotu E. Deadly lack of security plagues Nigeria as Buhari seeks re-election. The New York Times. https://www.nytimes.com/2018/08/15/world/africa/nigeria-zamfara-violence-buhari.html. Published August 15, 2018. Accessed July 3, 2019.

[35] Nugent C. Land conflict has long been a problem in Nigeria. Here’s how climate change Is making it worse. Time. Published online June 28, 2018. Accessed July 3, 2019. https://time.com/5324712/climate-change-nigeria/.

[36] UNHCR. Protection monitoring report Katsina, Sokoto and Zamfara; 2021. https://data2.unhcr.org/en/documents/download/86426.

[37] Sunday O. Nigeria farmers form vigilante groups to confront bandits. Al Jazeera. https://www.aljazeera.com/indepth/features/nigeria-farmers-form-vigilante-groups-confront-bandits-190514064544456.html. Published May 15, 2019. Accessed July 3, 2019.

[38] Mohammed A, Abdullahi M. Armed banditry and socio-economic development in Zamfara State: the assessment. EPRA Int J Res Dev. 2021; 6(12). DOI: 10.36713/epra2016

[39] Ahmed K. How my home town became a bloodbath that no-one cares about.https://www.bbc.com/news/world-africa-44126529. Published May 19, 2018. Accessed July 3, 2019.

[40] Campbell J. Farmer herder clashes in had follow familiar pattern. Council on Foreign Relations. Published May 21, 2019. Accessed July 3, 2019. https://www.cfr.org/blog/farmer-herder-clashes-chad-follow-familiar-pattern.

[41] UNEP. Environmental cooperation as a pathway to resolve Nigeria’s deadly farmer-herder conflicts. United Nations Environment Programme; 2018. Accessed July 3, 2019. http://www.unenvironment.org/news-and-stories/story/environmental-cooperation-pathway-resolve-nigerias-deadly-farmer-herder.

[42] Idris A. Children at risk in Nigeria due to lead poisoning from gold mines. Published online June 6, 2019. Accessed July 3, 2019. https://www.aljazeera.com/news/2019/06/children-risk-nigeria-due-lead-poisoning-gold-mines-190606083303551.html.

[43] MSF. NIGERIA: On the run from violence by MSF. Medecins Sans Frontieres. Published 2021. Accessed July 8, 2021. https://msf.exposure.co/nigeria-on-the-run-from-violence#!.

[44] Idris A. Will Nigeria crackdown on crime in Zamfara state work? Published online April 8, 2019. Accessed July 3, 2019. https://www.aljazeera.com/news/2019/04/nigeria-crackdown-crime-zamfara-state-work-190408174348699.html.

[45] MSF. Killings, looting and abductions in Zamfara State. Project Update. Published December 9, 2020. Accessed December 13, 2021. https://www.msf.org/nigeria-killings-looting-and-abductions-zamfara-state.

[46] Munshi N. Instability in the Sahel: how a jihadi gold rush is fuelling violence in Africa. Financial Times. https://www.ft.com/content/8ff4c2ca-7ac3-4f3b-96ba-6fb74bbb60d5. Published June 27, 2021. Accessed July 8, 2021.

[47] The World Bank. Artisanal and small-scale mining. Artisanal and small-scale mining. Published November 21, 2013. Accessed July 2, 2019. https://www.worldbank.org/en/topic/extractiveindustries/brief/artisanal-and-small-scale-mining.

[48] WHO. Artisanal and small-scale gold mining and health. World Health Organization; 2016. Accessed July 23, 2019. https://apps.who.int/iris/rest/bitstreams/1034034/retrieve.

[49] Calain P. What is the relationship of medical humanitarian organisations with mining and other extractive industries? PLoS Med. 2012; 9(8): e1001302. DOI: 10.1371/journal.pmed.1001302 22952440 PMC3429375

[50] Auty RM. Sustaining development in mineral economies: The resource curse thesis. Routledge; 1993.

[51] Okoh G, Hilson G. Poverty and livelihood diversification: Exploring the linkages between smallholder farming and artisanal mining in rural Ghana. J Int Dev. 2011; 23(8): 1100–1114. DOI: 10.1002/jid.1834

[52] Rittel HWJ, Webber MM. Dilemmas in a general theory of planning. Policy Sci. 1973; 4(2): 155–169. DOI: 10.1007/BF01405730

[53] Barnett J, Adger WN. Climate change, human security and violent conflict. Polit Geogr. 2007; 26(6): 639–655. DOI: 10.1016/j.polgeo.2007.03.003

[54] Zhang DD, Zhang J, Lee HF, He Y-q. Climate change and war frequency in Eastern China over the last millennium. Hum Ecol. 2007; 35(4): 403–414. DOI: 10.1007/s10745-007-9115-8

[55] Zhang DD, Brecke P, Lee HF, He YQ, Zhang J. Global climate change, war, and population decline in recent human history. Proc Natl Acad Sci U S A. 2007; 104(49): 19214–19219. DOI: 10.1073/pnas.0703073104 18048343 PMC2148270

[56] Ramsay JD, O’Sullivan TM. There’s a pattern here: The case to integrate environmental security into homeland security strategy. Homel Secur Aff. 2013; 9(6). Accessed July 21, 2019. https://commons.erau.edu/db-security-studies/10.

[57] United Nations Secretary General. Remarks to the security council on the impact of climate change on international peace and security. United Nations Secretary-General. Published July 20, 2011. Accessed June 21, 2019. https://www.un.org/sg/en/content/sg/speeches/2011-07-20/remarks-security-council-impact-climate-change-international-peace.

[58] Theisen OM, Holtermann H, Buhaug H. Climate wars? Assessing the claim that drought breeds conflict. Int Secur. 2011; 36(3): 79–106. DOI: 10.1162/ISEC_a_00065

[59] Buhaug H. Climate not to blame for African civil wars. Proc Natl Acad Sci. 2010; 107(38): 16477–16482. DOI: 10.1073/pnas.1005739107 20823241 PMC2944737

[60] Buhaug H, Benjaminsen TA, Sjaastad E, Theisen OM. Climate variability, food production shocks, and violent conflict in Sub-Saharan Africa. Environ Res Lett. 2015; 10(12): 125015. DOI: 10.1088/1748-9326/10/12/125015

[61] Theisen OM. Climate clashes? Weather variability, land pressure, and organized violence in Kenya, 1989–2004. J Peace Res. 2012; 49(1): 81–96. DOI: 10.1177/0022343311425842

[62] Devlin C, Hendrix CS. Trends and triggers redux: Climate change, rainfall, and interstate conflict. Polit Geogr. 2014; 43: 27–39. DOI: 10.1016/j.polgeo.2014.07.001

[63] Raleigh C, Choi HJ, Kniveton D. The devil is in the details: An investigation of the relationships between conflict, food price and climate across Africa. Glob Environ Change. 2015; 32: 187–199. DOI: 10.1016/j.gloenvcha.2015.03.005 28149004 PMC5268344

[64] Levy BS, Sidel VW. Collective violence caused by climate change and how it threatens health and human rights. Health Hum Rights. 2014; 16(1): 32–40. 25474608

[65] Busby JW, Cook KH, Vizy EK, Smith TG, Bekalo M. (PDF) Identifying hot spots of security vulnerability associated with climate change in Africa. Clim Change. 2014; 124: 717–731. DOI: 10.1007/s10584-014-1142-z

[66] Hsiang SM, Meng KC, Cane MA. Civil conflicts are associated with the global climate. Nature. 2011; 476(7361): 438–441. DOI: 10.1038/nature10311 21866157

[67] UNEP. Renewable resources and conflict. United Nations Environment Programme; 2012. Accessed July 2, 2019. https://reliefweb.int/report/world/toolkit-and-guidance-preventing-and-managing-land-and-natural-resources-conflict.

[68] Humphreys M, Sachs JD, Stiglitz JE. Escaping the Resource Curse. Columbia University Press; 2007.

[69] Lujala P, Rustad SA. High-Value Natural Resources and Post-Conflict Peacebuilding. Earthscan; 2012. DOI: 10.4324/9781849775786

[70] Dorner U, Franken G, Liedtke M, Sievers H. Artisanal and small-scale mining (ASM). In: Polinares: EU Policy on Natural Resources. CEPMLP, University of Dundee; 2012. Accessed July 23, 2019. http://pratclif.com/2015/mines-ressources/polinares/chapter7.pdf.

[71] Ocner MJ. Dirty gold is the new cocaine in Colombia — and it’s just as bloody. Miami Herald. Accessed July 8, 2021. https://www.miamiherald.com/news/nation-world/world/americas/colombia/article194188034.html.

[72] Shen L, Gunson AJ. The role of artisanal and small-scale mining in China’s economy. J Clean Prod. 2006; 14(3): 427–435. DOI: 10.1016/j.jclepro.2004.08.006

[73] Crawford A. Digging out of conflict: Can artisanal mining support peacebuilding? Published online September 27, 2017. Accessed June 23, 2019. https://www.iisd.org/blog/digging-out-conflict-can-artisanal-mining-support-peacebuilding.

[74] Kawamoto K. Diamonds in war, diamonds for peace: Diamond sector management and kimberlite mining in Sierra Leone. In: High-Value Natural Resources and Post-Conflict Peace Buliding. Earthscan; 2012; 121–145. https://www.eli.org/sites/default/files/121-145_kawamoto.pdf.

[75] AMDC. Policy brief: ASM and climate change. African Mining Development Centre

[76] Debrah AA, Watson I, Quansah DPO. Comparison between artisanal and small-scale mining in Ghana and South Africa: lessons learnt and ways forward. J South Afr Inst Min Metall. 2014; 114(11): 913–921.

[77] Geenen S. A dangerous bet: The challenges of formalizing artisanal mining in the Democratic Republic of Congo. Resour Policy. 2012; 37(3): 322–330. DOI: 10.1016/j.resourpol.2012.02.004

[78] Spiegel S, Keane S, Metcalf S, Veiga M. Implications of the Minamata Convention on Mercury for informal gold mining in Sub-Saharan Africa: from global policy debates to grassroots implementation? Environ Dev Sustain. 2015; 17(4): 765–785. DOI: 10.1007/s10668-014-9574-1

[79] Wilson ML, Renne E, Roncoli C, Agyei-Baffour P, Yamoah Tenkorang E. Integrated assessment of artisanal and small-scale gold mining in Ghana — part 3: Social sciences and economics. Int J Environ Res Public Health. 2015; 12(7): 8133–8156. DOI: 10.3390/ijerph120708133 26184277 PMC4515713

[80] IGF. Global trends in artisanal and small-scale mining (ASM): A review of key numbers and issues. Intergovernmental Forum on Mining, Minerals, Metals and Sustainable Development; 2017. Accessed July 2, 2019. https://www.iisd.org/sites/default/files/publications/igf-asm-global-trends.pdf.

[81] Lewis D, McNeill R, Shabalala Z. Gold worth billions is smuggled out of Africa. Reuters. https://www.reuters.com/investigates/special-report/gold-africa-smuggling/. Published April 24, 2019. Accessed June 23, 2019.

[82] Smith NM, Ali S, Bofinger C, Collins N. Human health and safety in artisanal and small-scale mining: an integrated approach to risk mitigation. J Clean Prod. 2016; 129: 43–52. DOI: 10.1016/j.jclepro.2016.04.124

[83] Mantz JW. Improvisational economies: Coltan production in the eastern Congo. Soc Anthropol. 2008; 16(1): 34–50. DOI: 10.1111/j.1469-8676.2008.00035.x

[84] Konkel Lindsey. A safer gold rush? Curbing mercury pollution in artisanal and small-scale gold mining. Environ Health Perspect. 127(11): 112001. DOI: 10.1289/EHP6417 PMC6927505 31763929

[85] Hilson G, Maconachie R. Formalising artisanal and small-scale mining: insights, contestations and clarifications. Area. 2017; 49(4): 443–451. DOI: 10.1111/area.12328

[86] Tschakert P, Singha K. Contaminated identities: Mercury and marginalization in Ghana’s artisanal mining sector. Geoforum. 2007; 38(6): 1304–1321. DOI: 10.1016/j.geoforum.2007.05.002

[87] Ruttinger L, Sharma V. Climate change and mining: A foreign policy perspective. adelphi; 2016. Accessed June 27, 2019. https://www.climate-diplomacy.org/publications/climate-change-and-mining-foreign-policy-perspective.

[88] IRP. Global resources outlook 2019: Natural resources for the future we want. Report of the International Resource Panel, United Nations Environment Programme; 2019. https://wedocs.unep.org/bitstream/handle/20.500.11822/27517/GRO_2019.pdf?sequence=3&isAllowed=y.

[89] WBG. The growing role of minerals and metals for a low carbon future. World Bank Group; 2017. Accessed January 2, 2020. http://documents.worldbank.org/curated/en/207371500386458722/pdf/117581-WP-P159838-PUBLIC-ClimateSmartMiningJuly.pdf.

[90] Ford JD, Pearce T, Prno J, et al. Canary in a coal mine: perceptions of climate change risks and response options among Canadian mine operations. Clim Change. 2011; 109(3): 399–415. DOI: 10.1007/s10584-011-0029-5

[91] Nelson J, Schuchard R. Adapting to climate change: A guide for the mining industry. BSR; 2011. https://www.bsr.org/reports/BSR_Climate_Adaptation_Issue_Brief_Mining.pdf.

[92] OCCIAR. Mining: In a changing climate. Ontario Cenre for Climate Impacts and Adaptation Resources Accessed July 23, 2019. http://climateontario.ca/doc/factsheets/Mining%20Factsheet%20--%20Final.pdf.

[93] Odell SD, Bebbington A, Frey KE. Mining and climate change: A review and framework for analysis. Extr Ind Soc. 2018; 5(1): 201–214. DOI: 10.1016/j.exis.2017.12.004

[94] Lahiri-Dutt K. Reframing the debate on informal mining. In: Between the Pick and the Plough. Anu Press; 2018. https://www.jstor.org/stable/j.ctt22h6r60. DOI: 10.22459/BPP.03.2018.01

[95] Sippl K, Selin H. Global policy for local livelihoods: Phasing out mercury in artisanal and small-scale gold mining. Environ Sci Policy Sustain Dev. 2012; 54(3): 18–29. DOI: 10.1080/00139157.2012.673452

[96] Steckling N, Tobollik M, Plass D, et al. Global burden of disease of mercury used in artisanal small-scale gold mining. Ann Glob Health. 2017; 83(2): 234–247. DOI: 10.1016/j.aogh.2016.12.005 28619398

[97] Seccatore J, Veiga M, Origliasso C, Marin T, De Tomi G. An estimation of the artisanal small-scale production of gold in the world. Sci Total Environ. 2014; 496: 662–667. DOI: 10.1016/j.scitotenv.2014.05.003 24867677

[98] UNEP. Land and conflict. United Nations Environment Programme; 2012. Accessed July 2, 2019. https://reliefweb.int/report/world/toolkit-and-guidance-preventing-and-managing-land-and-natural-resources-conflict.

[99] Maconachie R. Re-agrarianising livelihoods in post-conflict Sierra Leone? Mineral wealth and rural change in artisanal and small-scale mining communities. J Int Dev. 2011; 23(8): 1054–1067. DOI: 10.1002/jid.1831

[100] Sovacool BK, Ali SH, Bazilian M, et al. Sustainable minerals and metals for a low-carbon future. Science. 2020; 367(6473): 30–33. DOI: 10.1126/science.aaz6003 31896708

[101] Sumaina K. Nigeria: VP fault current efforts to tackle lead poisoning. This Day (Lagos). https://allafrica.com/stories/201806280523.html. Published June 28, 2018. Accessed July 8, 2019.

[102] ATSDR. Toxicological profile for mercury. US Department of Health and Human Services, Public Health Service: Agency for Toxic Substances and Disease Registry; 1999. Accessed November 25, 2019. https://www.atsdr.cdc.gov/toxprofiles/tp46.pdf.

[103] Esdaile LJ, Chalker JM. The mercury problem in artisanal and small-scale gold mining. Chem Weinh Bergstr Ger. 2018; 24(27): 6905–6916. DOI: 10.1002/chem.201704840 PMC5969110 29314284

[104] Hentschel T, Hruschka F, Priester M. Global report on artisanal & small-scale mining. Mining, Minerals and Sustainable Development; 2002. Accessed July 23, 2019. https://pubs.iied.org/pdfs/G00723.pdf.

[105] UNEP. Minamata Convention on Mercury: text and annexes. Accessed July 8, 2021. http://mercuryconvention.org/Convention/Text/tabid/3426/language/en-US/Default.aspx.

[106] Telmer KH, Veiga MM. World emissions of mercury from artisanal and small scale gold mining. In: Mason R, Pirrone N, (eds.), Mercury Fate and Transport in the Global Atmosphere: Emissions, Measurements and Models. Springer US; 2009: 131–172. DOI: 10.1007/978-0-387-93958-2_6

[107] de Lacerda L. Updating global Hg emissions from small-scale gold mining and assessing its environmental impacts. Environ Geol. 2003; 43(3): 308–314. DOI: 10.1007/s00254-002-0627-7

[108] Basu N, Clarke E, Green A, et al. Integrated assessment of artisanal and small-scale gold mining in Ghana--part 1: human health review. Int J Environ Res Public Health. 2015; 12(5): 5143–5176. DOI: 10.3390/ijerph120505143 25985314 PMC4454960

[109] Perez C, Jones EM, Kristjanson P, et al. How resilient are farming households and communities to a changing climate in Africa? A gender-based perspective. Glob Environ Change. 2015; 34: 95–107. DOI: 10.1016/j.gloenvcha.2015.06.003

[110] Watts N, Amann M, Arnell N, et al. The 2019 report of The Lancet countdown on health and climate change: ensuring that the health of a child born today is not defined by a changing climate. The Lancet. 2019; 394(10211): 1836–1878. DOI: 10.1016/S0140-6736(19)32596-6 PMC7616843 31733928

[111] Buss D, Rutherford B, Stewart J, et al. Gender and artisanal and small-scale mining: implications for formalization. Extr Ind Soc. 2019; 6(4): 1101–1112. DOI: 10.1016/j.exis.2019.10.010

[112] Bob U, Potgieter C, Perry E. Environmental conflicts and women’s vulnerability in Africa. ACCORD. Published October 26, 2010. Accessed November 22, 2019. https://www.accord.org.za/ajcr-issues/%ef%bf%bcenvironmental-conflicts-and-womens-vulnerability-in-africa/.

[113] UNOCHA. Global humanitarian overview 2019. United Nations Office for the Coordination of Humanitarian Affairs; 2018. Accessed November 22, 2019. https://www.unocha.org/sites/unocha/files/GHO2019.pdf.

[114] Werthmann K. Working in a boom-town: Female perspectives on gold-mining in Burkina Faso. Resour Policy. 2009; 34(1): 18–23. DOI: 10.1016/j.resourpol.2008.09.002

[115] Rustad SA, Østby G, Nordås R. Artisanal mining, conflict, and sexual violence in Eastern DRC. Extr Ind Soc. 2016; 3(2): 475–484. DOI: 10.1016/j.exis.2016.01.010

[116] Grigg J. Environmental toxins; their impact on children’s health. Arch Dis Child. 2004; 89(3): 244–250. DOI: 10.1136/adc.2002.022202 14977703 PMC1719840

[117] ATSDR. Toxicological profile for lead. Published online August 2007. Accessed December 12, 2019. https://www.atsdr.cdc.gov/ToxProfiles/tp.asp?id=96&tid=22.

[118] Landrigan PJ, Landrigan MM. Children and Environmental Toxins: What Everyone Needs to Know®. Oxford University Press; 2018. DOI: 10.1093/wentk/9780190662646.001.0001

[119] Bazillier R, Girard V. The gold digger and the machine.: Evidence on the distributive effect of the artisanal and industrial gold rushes in Burkina Faso. J Dev Econ. 2020; 143: 102411. DOI: 10.1016/j.jdeveco.2019.102411

[120] Maier RM, Díaz-Barriga F, Field JA, Hopkins J, Klein B, Poulton MM. Socially responsible mining: the relationship between mining and poverty, human health and the environment. Rev Environ Health. 2014; 29(1–2): 83–89. DOI: 10.1515/reveh-2014-0022 24552962 PMC4739650

[121] Hambling T, Weinstein P, Slaney D. A review of frameworks for developing environmental health indicators for climate change and health. Int J Environ Res Public Health. 2011; 8(7): 2854–2875. DOI: 10.3390/ijerph8072854 21845162 PMC3155333

[122] World Bank. Climate-smart mining: Minerals for climate action; 2018. Accessed July 8, 2021. https://www.worldbank.org/en/news/infographic/2019/02/26/climate-smart-mining.

[123] Slack JF, Kimball BE, Shedd KB. Cobalt, chapter F. In: Critical Mineral Resources of the United States—Economic and Environmental Geology and Prospects for Future Supply. US Geological Survey; 2017. Accessed July 8, 2020. DOI: 10.3133/pp1802F

[124] BBC. DR Congo country profile. BBC News. https://www.bbc.com/news/world-africa-13283212. Published February 4, 2021. Accessed July 8, 2021.

[125] Abdullahi M. The impact of armed banditry and human displacement on sustainable human development in Zamfara State. Zamfara J Polit Dev. 2021; 2(1). Accessed December 13, 2021. https://zjpd.com.ng/index.php/zjpd/article/view/29.

[126] Edwards DP, Sloan S, Weng L, Dirks P, Sayer J, Laurance WF. Mining and the African Environment. Conserv Lett. 2014; 7(3): 302–311. DOI: 10.1111/conl.12076

[127] NIC. North Africa: The impact of climate change to 2030. National Intellegence Council; 2009. Accessed July 2, 2019. https://www.dni.gov/files/documents/2009%20Conference%20Report_North%20Africa_The%20Impact%20of%20Climate%20Change%20to%202030.pdf.

[128] UNICEF. Generation 2030 Africa 2.0. United Nations Children’s Fund; 2017. Accessed July 3, 2019. https://www.unicef.org/publications/files/Generation_2030_Africa_2.0.pdf.

[129] UN. World population prospects 2019: Ten key findings. United Nations, Department of Economic and Social Affairs, Population Division; 2019. https://population.un.org/wpp/Publications/Files/WPP2019_10KeyFindings.pdf.

[130] Hallmark T. Oil and violence In the Niger delta isn’t talked about much, but it has a global impact. Forbes. Published online February 13, 2017. Accessed June 23, 2019. https://www.forbes.com/sites/uhenergy/2017/02/13/oil-and-violence-in-the-niger-delta-isnt-talked-about-much-but-it-has-a-global-impact/.

[131] Okonta I, Douglas O. Where Vultures Feast: Shell, Human Rights, and Oil in the Niger Delta. Sierra Club Books; 2001.

[132] MSF. Who we are: We are Médecins Sans Frontières. Médecins Sans Frontières (MSF) International. Accessed June 23, 2019. https://www.msf.org/who-we-are.

